# Vitamin D exposure and Risk of Breast Cancer: a meta-analysis

**DOI:** 10.1038/s41598-018-27297-1

**Published:** 2018-06-13

**Authors:** Nuria Estébanez, Inés Gómez-Acebo, Camilo Palazuelos, Javier Llorca, Trinidad Dierssen-Sotos

**Affiliations:** 1 University of Cantabria – IDIVAL, Santander, Spain; 2 CIBER Epidemiología y Salud Pública (CIBERESP), Madrid, Spain

## Abstract

The relationship between vitamin D and breast cancer is still controversial. The present meta-analysis examines the effects of the 25(OH)D, 1,25(OH)2D and vitamin D intake on breast cancer risk. For this purpose, a PubMed, Scopus and Web of Science-databases search was conducted including all papers published with the keywords “breast cancer” and “vitamin D” with at least one reported relative risk (RR) or odds ratio (OR). In total sixty eight studies published between 1998 and 2018 were analyzed. Information about type of study, hormonal receptors and menopausal status was retrieved. Pooled OR or RR were estimated by weighting individual OR/RR by the inverse of their variance Our study showed a protective effect between 25 (OH) D and breast cancer in both cohort studies (RR = 0.85, 95%CI:0.74–0.98) and case-control studies (OR = 0.65, 95%CI: 0.56–0.76). However, analyzing by menopausal status, the protective vitamin D – breast cancer association persisted only in the premenopausal group (OR = 0.67, 95%CI: 0.49–0.92) when restricting the analysis to nested case-control studies. No significant association was found for vitamin D intake or 1,25(OH)2D. Conclusion: This systematic review suggests a protective relationship between circulating vitamin D (measured as 25(OH) D) and breast cancer development in premenopausal women.

## Introduction

Breast cancer is an important public health problem in developed countries as it is one of the most common cancers, being the most if only the female population is considered^1^. The incidence is decreasing every year, which is partly due to early detection programs^2^.

In the last decades, cellular *in vitro* experiments and *in vivo* models have evaluated the role of vitamin D in the development of breast cancer, finding a protective anticancer role of 1,25(OH)D3^3^. It has been demonstrated that treating breast cancer cells with 1,25(OH)D3 induces two beneficial effects: an anti-proliferative effect^4^ and a pro-apoptotic effect^5,6^. The former is linked to the suppression of growth stimulatory signals and the potentiation of growth inhibitory signals, whilst the second one is explained by the bcl-2 family proteins. The interaction between vitamin D and its receptors induces an increase in the expression of pro-apoptotic family member (bax and bak protein) and simultaneously a decrease of anti-apoptotic (bcl-2/bcl-XL)^6^. In addition, the breast tissue contains the 1-α-hydroxylase, allowing for the generation of the active vitamin D metabolite (1,25 dihydroxyvitamin D) from the circulating precursor (25 hydroxyvitamin D). As vitamin D receptors are found in the breast^6^, an autocrine role of vitamin D has been suggested^7^.

Despite this biological background, literature shows inconsistent results^8–16^ (Table 1). Several additional observational studies have appeared since the last meta-analysis publication (including articles until 2013). The main purpose of the present meta-analysis is to update the relationship between vitamin D exposure and breast cancer risk by adding the studies published more recently. Thus sixty-eight observational studies: thirty of these were case-control, twenty-one were nested case-control and the remaining were cohort studies.

**Table 1:** RR of breast cancer and vitamin D in previous meta-analysis.

Source	Type of vitamin D	Number of included studies	Type of included studies	RR (95%IC)
Bauer SR *et al*. (2013)	25(OH)D	9	Cohort & nested case-control studies	0.9 (0.97–1.00)
Chen P *et al*. (2010)	25(OH)D	21	Case control, cohort, & cross-sectional studies	0.55 (0.38–0.80)
	Intake of vitamin D			0.91 (0.85–0.97)
	1,25(OH)2D			0.99 (0.68–1.44)
Chen P *et al*. (2013)	25(OH)D	21	Nested case-control & retrospective studies	0.86 (0.75–1.00)
			Population based case control studies	0.35 (0.24–0.52)
			Hospital based case-control studies	0.08 (0.08–0.33)
Gandini S *et al*. (2011)	25(OH)D	10	Case-control	0.83 (0.79–0.87)
			Nested case-control & cohort studies	0.97 (0.92–1.03)
Gissel T *et al*. (2008)	Intake of vitamin D	6	Cross sectional, Case-control, cohort & r&omized-control trials	0.98 (0.93–1.03)
Kim Y and Je Y. (2014)	Intake of vitamin D	24	Cohort & nested case-control studies	0.95 (0.88–1.01)
	25(OH)D			0.92 (0.83–1.02)
Wang D *et al*. (2013)	25(OH)D	14	Cohort & nested case-control studies	0.84 (0.75–0.95)
Mohr SB *et al*. (2011)	25(OH)D	11	All	0.61 (0.47–0.80)
			Case-control studies	0.87 (0.77–0.99)
			Nested case-control studies	0.41 (0.31–0.56)
Yin L *et al*. (2010)	25(OH)D	9	All	0.73 (0.60–0.88)
			Nested case-control	0.92 (0.82–1.04)
			Case- control	0.59 (0.48–0.73)

## Methods

### Search strategy

Firstly, the following inclusion criteria were defined: we looked for cohort or case-control studies performed in humans, which reported, at least, one relative risk (RR) or odds ratio (OR) with confidence interval at 95%. (95% CI)

**Figure 1:**
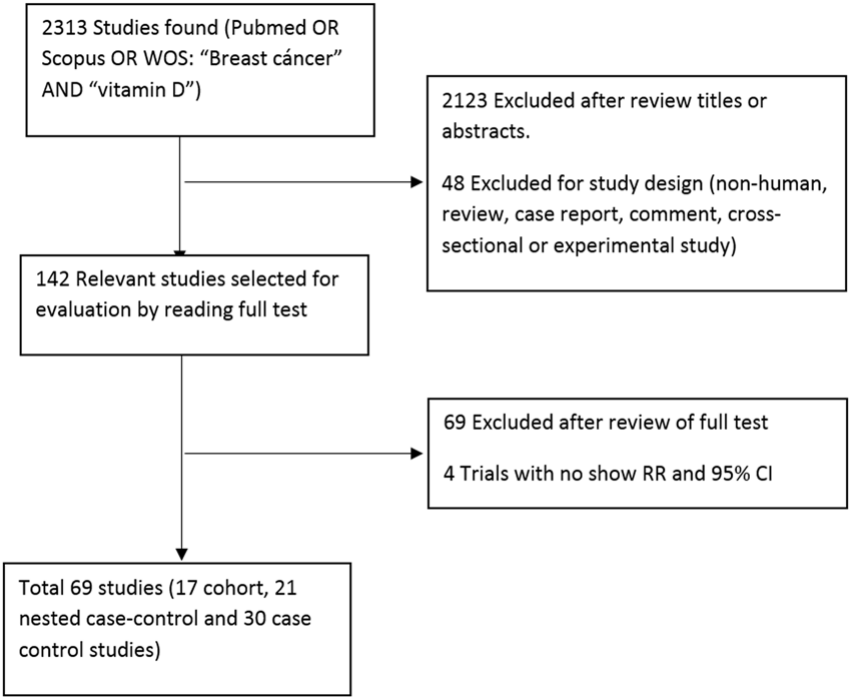
Flowchart which describes the methodology of selection of the articles.

We began our search in Pub-Med, Scopus and Web of Science database using “breast cancer” and “vitamin D” as keywords, finding 2313 articles. After having read the title and abstract, 2123 articles that did not meet the above criteria were eliminated. Next, we carried out a more exhaustive and complete reading, which allowed us to reject another additional 69 articles (Fig. 1). Finally, sixty eight studies meeting our inclusion criteria were identified: fifty one case-control^10,17–65^ and seventeen cohort studies^65–81^. Tables 2 and 3 summarize the main characteristics of the included articles.

**Table 2:** Studies included in our meta-analyses of blood 25-hydroxyvitamin D and breast cancer risk.

Nested Case-Control	Country	Exposition	Group	OR 95% CI	No. of participants	Age at baseline^a^	Follow-up period	Upper vs lower cut off levels	Adjusted by Time of blood draw
Almquist M *et al*.(2010)^£,¥,§,φ^	Sweden	25(OH)D3	All	0.99 (0.72–1.36)	1524	57 years	1991–2006	≥106 vs ≤70 ng/mL	Yes
		25(OH)D3 + D2	All	1.01 (0.73–1.40)				≥107 vs ≤71 ng/mL	
		25(OH)D3	PRE	1.58 (0.77–3.25)				≥106 vs ≤70 ng/mL	
			POST	0.88 (0.60–1.28)				≥107 vs ≤71 ng/mL	
		25(OH)D3 + D2	PRE	1.74 (0.84–3.60)				≥106 vs ≤70 ng/mL	
			POST	0.88 (0.60–1.29)				≥107 vs ≤71 ng/mL	
Amir E *et al*. (2012)^£^	Canada	25(OH)D	All	0.86 (0.62–1.21)	1087	53.6 years	1992–1997	≥34.4 vs <12 ng/mL	No
Bertone-Johnson ER *et al*. (2005)^£,¥,§^	USA	25(OH)D	All	0.73 (0.49–1.07)	1425	52.7 cases 57.1 controls	1989–1996	≥48 vs <20 ng/mL	No
		1,25(OH)D	All	0.76 [0.52–1.11]				≥38.2 vs <28.5 ng/mL	
Chlebowski RT *et al*. (2008)^€,£,§,ǂ,$^	USA	25(OH)D	POST	0.82 (0.60–1.12)	2134	50–79 years	1995–2002	≥27.04 vs <12.96 ng/mL	Yes
Deschasaux M *et al*. (2016)^£, ¥,ǂ,φ^	France	25(OH)D	All	0.98 (0.60–1.61)	699	49.3 cases 49.1 controls	1994–2007	≥23.5 vs <11.4 ng/mL	Yes
Eliassen AH *et al*. (2011)^£,¥^	USA	25(OH)D	All	1.20 (0.88–1.63)	1827	45 cases 44.9 controls	1996–2007	≥30.6vs <18.4 ng/mL	No
			ER+	1.21 (0.84–1.75)					
			ER−	1.31 (0.63–2.74)					
Eliassen AH *et al*.(2016)^£,¥^	USA	25(OH)D	All	0.84 (0.58–1.21)	3012	56.7 cases 56.8 controls	1989–2010	≥32.7 ng/ml vs <17.5	No
			ER+	0.89 (0.74–1.08)				≥30 ng/ml vs <30	
			ER−	0.87 (0.63–1.20)					
Engel P *et al*. (2010)^€,£, ¥, ǂ^	France	25(OH)D	All	0.73 (0.55–0.96)	1908	56.9 years	1995–2005	>27 vs <19.8 ng/ml	Yes
			PRE	0.37 (0.12–1.15)					
			POST	0.80 (0.60–1.07)					
Freedman M *et al*. (2008)^€,£,¥¥,§^	USA	25(OH)D	POST	1.04 (0.72–1.51)	2010	55–74 years	1993–2005	33.7 vs 18.3 ng/mL	Yes
Hiatt RA *et al*. (1998)^¥,φ^	USA	1,25(OH)2D	All	1.00 (0.20–3.40)	192	>55 years	1980–1991	≥51 vs <32 pg/ml	No
Kim Y *et al*. (2014)^£,¥,$^	USA	25(OH)D	White	0.13 (0.03–0.71)	1414	68.5 cases 68.4 controls	2001–2006	>0 vs 0 ng/mL	Yes
			African-american	1.35 (0.65–2.78)					
			Hawaian	1.35 (0.23–7.69)					
			Japanese	1.04 (0.51–2.13)					
			Latino	1.11 (0.51–2.44)					
Kühn T *et al*. (2013)^£,¥,ǂ,φ^	Europe	25(OH)D	All	1.07 (0.85–1.36)	2782	50.7 years	1992–2006	>63 vs ≤39.3nmol/L	No
			ER+	0.97 (0.67–1.38)					
			ER−	0.97 (0.66–1.42)					
McCullough ML *et al*.(2009)^£,¥,$^	USA	25(OH)D	All	1.09 (0.70–1.68)	1032	69.5 cases 69.4 controls	1998–2005	>76.2vs <36.7 nmol/ml	Yes
			ER+	1.15 (0.80–1.65)				>64.2 vs <45.9 nmol/ml	
			ER−	0.95 (0.43–2.06)					
Mohr SB *et al*. (2013)^$^	USA	25(OH)D	All	0.84(0.56–1.25)	1200	39.6 years	1994–2009	≥35.2 vs ≤14.9 ng/mL	No
Neuhouser ML *et al*. (2012)^£,ǂ^	USA	25(OH)D	POST	0.94 (0.70–1.28)	2160	50–79 years	1994–2005	≥25.96vs ≤14.68 ng/mL	No
Rejnmark L *et al*. (2009)^#^	Denmark	25(OH)D	All	0.52 (0.32–0.85)	562	58 years	2003–2007	>33.6 vs <24 ng/mL	No
			PRE	0.38 (0.15–0.97)					
			POST	0.71 (0.38–1.30)					
Scarmo S *et al*. (2013)^£,¥,§^	USA&Sweden	25(OH)D	All	0.94 (0.76–1.16)	4525	34–69 years	1985–2007 1995–2010	N.A. (Quintiles)	No
			PRE	0.67 (0.48–0.92)					
			POST	1.21 (0.92–1.58)					
Shirazi L *et al*. (2016)^€,£, ¥,§^	Sweden	25(OH)D3	All	0.97 (0.75–1.25)	1520	46–73 years	1991–1996/2006	≥98nmol/L vs ≤76nmol/L	Yes
Wang J *et al*. (2014)^£,¥^	USA	25(OH)D	All	0.95 (0.67–1.36)	1168	45 years		>= 5.59 vs <3.76nmol/L	No
**Case-Control**	**Country**	**Exposition**	**Group**	**OR 95% CI**	**No. of participants**	**Age at baseline**	**Follow-up period**	**Upper cut off levels**	
Abbas S *et al*. (2009)^£,¥,φ^	Germany	25(OH)D	PRE	0.45 (0.29–0.70)	884	42.1 cases 41.6 controls	1992–1995	≥60 vs <30nmol/L	Yes
			ER+	0.56 (0.31–1.00)					
			ER−	0.40 (0.20–0,81)					
Abbas S *et al*. (2008)^£,¥,§^	Germany	25 (OH)D	POST	0.31 (0.24–0.42)	2759	63.6 cases 63.5 controls	2001–2005	> = 75 vs <30nmol/L	Yes
Alipour S *et al*. (2014)^€, ¥^	Iran	25 (OH)D	All	0.33 (0.12–0.91)	500	44.2 cases 43.2 controls	N.A.	>35 ng/ml vs <12.5 ng/ml	No
Bilinski K *et al*. (2012) ^€,φ^	Australia	25(OH)D	All	0.43 (0.23–0.77)	1066	55.4 cases 55.5 controls	2008–2011	≥75nmol/L vs <25nmol/mL	Yes
			<50years	0.29 [0.08–1]					
			≥50 years	0.45 [0.23–0.71]					
Chen P *et al*. (2013)^€, ¥,§^	China	25(OH)D	All	0.11 (0.07–1.17)	1173	53.0 cases 55.3 controls	2005–2008	>17.9 ng/ml vs <10.4 ng/ml	Yes
Colagar AH *et al*. (2015)^#^	Iran	25(OH)D	All	0.26 (0.13–0.50)	261	48.7 cases 47.0 controls	2009–2013	≥16 vs <9 ng/mL	No
Crew KD *et al*. (2009) ^€,£,¥,§,ǂ,$^	USA	25(OH)D	All	0.56 (0.41–0.78)	2101	58.6 cases 56.1 controls	1996–1997	≥40 vs <20 ng/mL	Yes
			PRE	0.83 [0.36–1.30]					
			POST	0.46 [0.09–0.83]					
Fedirko V *et al*. (2012)^£,¥¥,§,ǂ,φ^	Mexico	25(OH)D3	All	0.53 (0.36–0.78)	2074	53.1 cases 51.3 controls	2004–2007	>25 vs ≤20 ng/mL	Yes
			PRE	0.40 (0.30–0.81)					
			POST	0.55 (0.33–0.90)					
Jamshidinaein Y *et al*. (2016)^£,§,φ,$^	Iran	25(OH)D	All	0.26 (0.12–0.59)	270	50.4 cases 50.0 controls	2013–2014	≥29.5 vs <10.30 ng/ml	Yes
			PRE	0.25 (0.09–0.69)					
			POST	0.42(0.15–1.17)					
Janowsky EC *et al*. (1999)^€^	USA	1,25(OH)2D	All	0.31 (0.17–0.59)	331	NA	1990–1991	≤34.6 vs>63.6pmol/ml	Yes
Lowe LC *et al*.(2005)^€^	UK	25(OH)D	All	0.17 (0.07–0.43)	358	58.0 cases 58.0 controls	1998–2003	≥150 vs ≤50 nM	Yes
Oliveira-Sediyama CM *et al*.(2016)^ǂ^	Brazil	25(OH)D	All	0.34 (0.16–0.71)	378	54.0 cases 47.5 controls	NA	≥20vs <20 ng/mL	No
Park S *et al*. (2015)^€,£, ¥,§^	Korea	25(OH)D	All	0.82 (0.75–0.90)	20767	50.7 cases 49.7 controls	2006–2012	≥20 vs <20 ng/mL	Yes
			PRE	0.84 (0.74–0.96)					
			POST	0.82 (0.73–0.93					
Sofi NY *et al*. (2016)^#^	India	25(OH)D	All	0.40 (0.14–1.11)	200	45.0 cases 46.0 controls	2014–2015	≥20 ng/mL vs <20 ng/mL	No
Sofi NY *et al*. (2018)^#^	India	25(OH)D	All	0.42 (0.20–0.83)	400	45.0 cases 47.0 controls	2015–2017	≥20 ng/mL vs <20 ng/mL	No
Yao S *et al*. (2011)^€,£,¥^	USA	25(OH)D	All	0.37 (0.27–0.51)	1153	NA	2003–2008	≥30 vs <20 ng/mL	Yes
			PRE	0.57 (0.34–0.93)					
			POST	0.29 (0.19–0.45)					
Yousef FM *et al*. (2013)^€,£,φ^	Saudi Arabia	25(OH)D	All	0.16 (0.07–0.42)	240	18–75 years	2009	≥20 vs <10 ng/mL	No
Ordoñez-Mena JM *et al*. (2016)^€,£,ǂ,φ^	Europe	25(OH)D	POST	0.73 (0.22–2.43)	252	> = 60 years	1992–2000	>50 vs <30 nmol/L	Yes
**Cohort**	**Country**	**Exposition**	**Group**	**RR 95% CI**	**Cases (No. of participants)**	**Age at baseline**	**Follow-up period**	**Upper cut off levels**	
Skaaby T *et al*. (2014)^£,ǂ,φ^	Denmark	25(OH)D	All	1.1 (0.7–1.71)	159 (5606)	18–71 years	1993–2008	N.A. (Quartiles)	Yes
O´Brien KM (2017) et al^€,£, ¥,§,ǂ,φ,$^	USA	25(OH)D	All	0.79 (0.63–0.98)	1600 (3422)	35–74 years	2003–2009	>38 vs <24.6 ng/mL	Yes
Ordonez-Mena JM *et al*. (2013)^€,£,ǂ,φ^	Germany	25(OH)D	All	1.08 (0.72–1.6)	137 (5261)	50–74 years	2000–2002	<30 vs >55 nmol/L*	No
Palmer JR *et al*. (2016)^€,£, ¥,§^	USA (African American Women)	25(OH)D	All	0.81 (0.68–0.96)	1454 (2856)	21–69 years	2012–2017	≥49 vs <21 ng	No
Ordonez-Mena JM *et al*. (2016)^€,£, ǂ,φ^	Germany	25(OH)D	POST	1.35 (0.38–2.27)	63 (4990)	63 years	2000–2002	>50 vs <30nmol/L	Yes
Ordonez-Mena JM *et al*. (2016)^€,£,ǂ,φ^	Norway	25(OH)D	POST	2.63 (0.82–8.33)	89 (2471)	62 years	1994–1995	>50 vs <30nmol/L	Yes

^a^Mean or range of age.

Adjusted by: ^€^age; ^£^BMI; ^¥^reproductive factors (menopausal status, age at menopause, age at menarche, parity, etc); ^§^hormone therapy; ^ǂ^physical activity; ^φ^educative or socioeconomic variables; ^$^race or sun exposure.

^#^Unadjusted.

Abbreviations: CI = confidence interval; POST = postmenopausal; PRE = premenopausal; OR = odds ratio; NA: Not available.

**Table 3:** Studies included in our meta-analyses of dietary or supplements vitamin D and breast cancer risk.

Case-Control	Country	Exposition	Group	OR (95% CI)	No. of participants	Age at baseline	Follow-up period	Upper vs lower cut off levels
Abbas S *et al*. (2007)^€,£,¥^	Germany	Dietary Vitamin D	PRE	0.50 (0.26–0.96)	944	41.7 cases 41.6 controls	1992–1995	≥200 vs <80 IU/day
Anderson LN *et al*. (2010)^€,¥,ǂ,φ^	Canada	Total vitamin D intake	All	0.99 (0.78–1.26)	6572	56 years	2002–2003	≥15 vs <2.5 mg/day
		Dietary Vitamin D		1.13 (0.88–1.45)				≥10 vs <2.5 mg/day
		Vitamin D supplement		0.76 (0.59–0.98)				≥10 vs 0 mg/day
Anderson LN *et al*. (2011)^€^	Canada	Vitamin D supplement	All	0.80 (0.60–1.08)	3616	56 years	2002–2003	>400 vs 0 IU/day
		Total Vitamin D intake		0.87 (0.71–1.06)				≥600 vs <200 IU/day
Bidgoli SA *et al*. (2014)^#^	Iran	Vitamin D supplement	PRE	0.89 (0.84–0.95)	176	36.5 cases 34.2 controls	2010–2012	Yes vs No
Jamshidinaein Y *et al*. (2016)^€,£,¥,§,φ^	Iran	Dietary vitamin D	All	0.38 (0.18–0.83)	270	50.4 cases 50 controls	2013–2014	NA (Quartile)
		Dietary vitamin D	PRE	0.39 (0.15–1.00)				
		Dietary vitamin D	POST	0.40 (0.15–1.12)				
		Total vitamin D intake	All	0.52 (0.25–1.14)				
		Total vitamin D intake	PRE	0.36 (0.13–1.06)				
		Total vitamin D intake	POST	0.70 (0.27–1.82)				
Kawase T *et al*. (2010)^£,¥,§,ǂ^	Japan	Dietary Vitamin D	All	0.76 (0.63–0.90)	5409	20–79	2001–2005	>6.66 vs <2 mg/day
			PRE	0.65 (0.50–0.86)				
			POST	0.83 (0.64–1.07)				
Lee MS *et al*. (2011)^€,£,¥,φ^	Taiwan	Dietary Vitamin D	All	0.57 (0.28–1.19)	400	52.5 cases 48.9 controls	2004–2005	>5 vs <2 mg/day
		Dietary Vitamin D	PRE	0.38 (0.14–0.98)				
		Dietary Vitamin D	POST	0.60 (0.20–1.69)				
		Total vitamin D intake	All	0.52 (0.25–1.07)				NA (Quartile)
		Total vitamin D intake	PRE	0.47 (0.18–1.23)				
		Total vitamin D intake	POST	0.68 (0.23–1.27)				
Levi F *et al*.(2001)^€,£,¥,φ^	Switzerland	Vitamin D supplement	All	1.43 (0.90–2.26)	731	23–74	1993–1999	≥2.7 vs <1.4 mg/day
Leung *et al*.(2016)^€^	China	Vitamin D supplement	All	0.78 (0.63–0.98)	323612	>18	2000–2011	≤15 DDD
Potischman N *et al*. (1999)^€,¥,§,φ^	USA	Dietary Vitamin D	All	0.98 (0.80–1.20)	2019	20–44	1990–1992	≥400 vs <0 IU
Rollison DE *et al*. (2012)^€,£,¥,§,ǂ^	USA	Dietary Vitamin D	All	1.35 (1.15–1.60)	4839	24–79	1999–2004	7.71 vs <3.06 mg/day
		Vitamin D supplement	All	0.79 (0.65–0.96)		24–79 years	1999–2004	0 vs>10 mg/day
Rossi M *et al*. (2009)^€,£,¥,§,φ^	Italy	Dietary Vitamin D	All	0.76 (0.58–1.00)	5157	55 years cases 56 controls	1991–1994	>3.57 vs ≤3.57 mg
			PRE	0.80 (0.64–0.99)				
			POST	0.78 (0.66–0.92)				
Salarabadi A *et al*. (2015)^#^	Iran	Vitamin D supplement	PRE	0.53 (0.14–1.96)	152	NA	2012–2014	Yes vs No
**Cohort**	**Country**	**Exposition**	**Group**	**RR (95% CI)**	**Cases/Total**	**Age at baseline**	**Follow-up period**	**Upper cut off levels**
John EM *et al*. (1999)^€,£,¥,ǂ,φ^	USA	Dietary vitamin D	All	0.85 (0.59–1.24)	190/5009	25–74	1971–1992	≥200 vs <100 IU/day
		Vitamin D supplement	All	0.89 (0.60–1.32)		25–74	1971–1993	Daily vs never
		Total vitamin D intake	All	0.86 (0.61–1.2)		25–74	1971–1994	≥200 or daily suppl vs <100 IU/day without daily suppl
Shin MH *et al*. (2002)^€,£,¥,ǂ^	USA	Total vitamin D intake	PRE	0.89 (0.68–1.15)	3482/88 691	46.7	1980–1996	>500 vs ≤150 IU/day
			POST	0.93 (0.8–1.08)				
		Dietary Vitamin D	PRE	0.84 (0.59–1.18)				
			POST	0.86 (0.7–1.05)				
Lin J *et al*. (2007)^€,£,¥,§,ǂ^	USA	Total vitamin D intake	PRE	0.65 (0.42–1)	1019/31487	55 (≥45)	1993–2003	≥548 vs <162 IU/d
			POST	1.30 (0.97–1.73)				
		Dietary vitamin D	PRE	1.02 (0.69–1.53)				≥319 vs <142 IU/d
			POST	1.22 (0.95–1.55)				
		Vitamin D supplement	PRE	0.76 (0.5–1.17)				≥400 vs 0 IU/d
			POST	0.87 (0.68–1.12)				
Robien K *et al*. (2007)^€,£,¥,§,φ^	EEUU	Vitamin D supplement	POST	0.89 (0.74–1.08)	2440/34321	61 (55–69)	1986–2004	≥800 IU/d vs No
		Dietary Vitamin D	POST	0.55 (0.24–1.22)				≥800 vs <400 IU/d
		Total vitamin D intake	POST	0.89 (0.77–1.03)				≥800 vs <400 IU/d
Kuper H *et al*. (2009)^€,£,¥,§,ǂ^	Sweden	Dietary vitamin D	All	0.90 (0.80–1.1)	848/41889	30–49	1991–2003	N.A. (Quartile)
Cadeau C *et al*. (2015) ^€,£,¥,§,ǂ^	France	Vitamin D supplement	All	1.10 (0.92–1.31)	2482/57403	40–65	1995–2008	Current vs never
			ER+	1.23 (1–1.51)		40–65	1995–2008	Current vs never
			ER−	0.93 (0.55–1.55)		40–65	1995–2008	Current vs never
Abbas S *et al*. (2013)^€,¥,§,ǂ,φ^	Europe	Dietary vitamin D	All	1.04 (0.94–1.14)	7760/319985	50.2	1992–2005	≥5.46 vs <1.85 mg/day
			PRE	1.07 (0.87–1.32)				≥5.46 vs <1.85 mg/day
			POST	1.02 (0.9–1.16)				≥5.46 vs <1.85 mg/day
McCullough ML *et al*. (2005)^€,¥,§,ǂ,φ^	USA	Total vitamin D intake	POST	0.94 (0.8–1.1)	2855/68567	50–74	1992–2001	>700 vs ≤100 IU/day
		Dietary vitamin D	POST	0.87 (0.75–1)				>300 vs ≤100 IU/day
Edvarsen K *et al*. (2011) ^€,£,¥,§^	Norway	Dietary vitamin D	All	1.07 (0.87–1.32)	948/41811	40–70	1997–2007	12.31 vs <3.99 mg/day
Frazier *et al*. (2004)^€,£,¥,§^	USA	Dietary vitamin D	All	0.92 (0.66–1.27)	838/47355	34–51	1989–1998	591 vs 159.6 IU/day
Engel P et al. (2011)^£,¥,§,ǂ^	France	Total vitamin D intake	All	0.94 (0.86–1.03)	2871/67721	41.8–72	1990–2008	>113 vs <80 IU/day
			PRE	1.03 (0.85–1.25)				
			POST	0.92 (0.86–1.03)				
**Nested Case-Control**	**Country**	**Exposition**	**Group**	**OR (95% CI)**	**No. of participants**	**Age at baseline**	**Follow-up period**	**Upper vs lower cut off levels**
Simard A *et al*. (1991)^#^	Canada	Dietary Vitamin D	All	2.79 (0.85–9.15)	430	40–59	1981–1983	>200 vs <50 IU/day
Kim Y *et al*. (2014)^£,¥,ǂ^	USA	Vitamin D supplement	White	1.29 (0.75–2.23)	1414	67.8	2001–2010	> = 16 ng/mL vs <16 ng/mL
			African-american	0.29 (0.12–0.70)				
			Hawaian	0.46 (0.16–1.34)				
			Japanese	1.32 (0.90–1.93)				
			Latino	0.85(0.46–1.56)				
			PRE	1.03 (0.85–1.25)				
			POST	0.92 (0.86–1.03)				

^a^*Mean or range of age*.

Adjusted by: ^€^age; ^£^BMI; ^¥^reproductive factors (menopausal status, age at menopause, age at menarche, parity, etc); ^§^hormone therapy; ^ǂ^physical activity; ^φ^educative or socioeconomic variables; ^$^race or sun exposure.

^#^Unadjusted.

*Abbreviations: CI* = *confidence interval; POST* = *postmenopausal; PRE* = *premenopausal;* OR = odds ratio*; NA: Not available*.

### Data extraction

The following step was to create a database to gather all relevant information extracted from each article: year of publication, author, journal, follow up, country, sample size, exposure levels, units of measure, data for the creation of the contingency table and RR/OR with 95% CI; as well as a section to assess the quality of the study using the STROBE scale^82^.

### Statistical analysis

Statistical analysis was performed separately for cohort and case-control studies. In the case control studies a sensitivity analysis was also carried-out including only nested case-control studies. We performed separate analyses for any type of vitamin D exposure reported in at least three studies: 25(OH)D, dietary intake of vitamin D, 1,25(OH)2D and vitamin D supplements.

The ways that doses or levels of vitamin D were reported in each individual article were not standardized across studies (for instance, some papers reported vitamin D levels in quartiles; others in tertiles, and so on), making it difficult to extract them in an analyzable form. Therefore, in order to provide a consistent criterion of comparability, we selected the OR or RR reported for the highest category compared to the lowest one.

Regarding the type of breast cancer, we analyzed all invasive breast cancers together, and breast cancer stratified according to the cancer estrogen receptor status and woman’s menopausal status. Pooled OR or RR were estimated by weighting individual OR/RR by the inverse of their variance. OR or RR heterogeneity was measured using Q and I^2^ statistics^83^. A fixed-effect model was preferred if the Q statistic was higher than 0.1 or I^2^ lower than 25%, indicating no relevant heterogeneity; a random-effect model was otherwise chosen^84^. The presence of small-study bias was explored with Rosenthal model and with Egger test^85^; due to the low sensitivity of Egger test, the cut-off was set at p = 0.1. Funnel plots^86^ were applied to detect publication bias.

An analysis of influence was performed via the re-estimation of pooled OR/RR by removing one study at a time. Studies that, when removed, strongly changed the OR/RR would be considered as highly influential. Results are displayed as forest plots showing OR/RR and their 95% confidence intervals for each individual study and for the pooled result. Cumulative meta-analyses were carried out to deem the stability of the OR/RR estimates. In order to do that, all studies considered were arranged from oldest to neweest. Then an OR/RR estimate was obtained for the two eldest studies; another for the three eldest, and so on, adding a study each time. Results are reported as forest plots.

All the statistical analyses were carried out with the package Stata 14/SE (Stata Corporation, College Station, TX, US).

## Results

### Relationship between 25(OH) D and breast cancer

**Figure 2:**
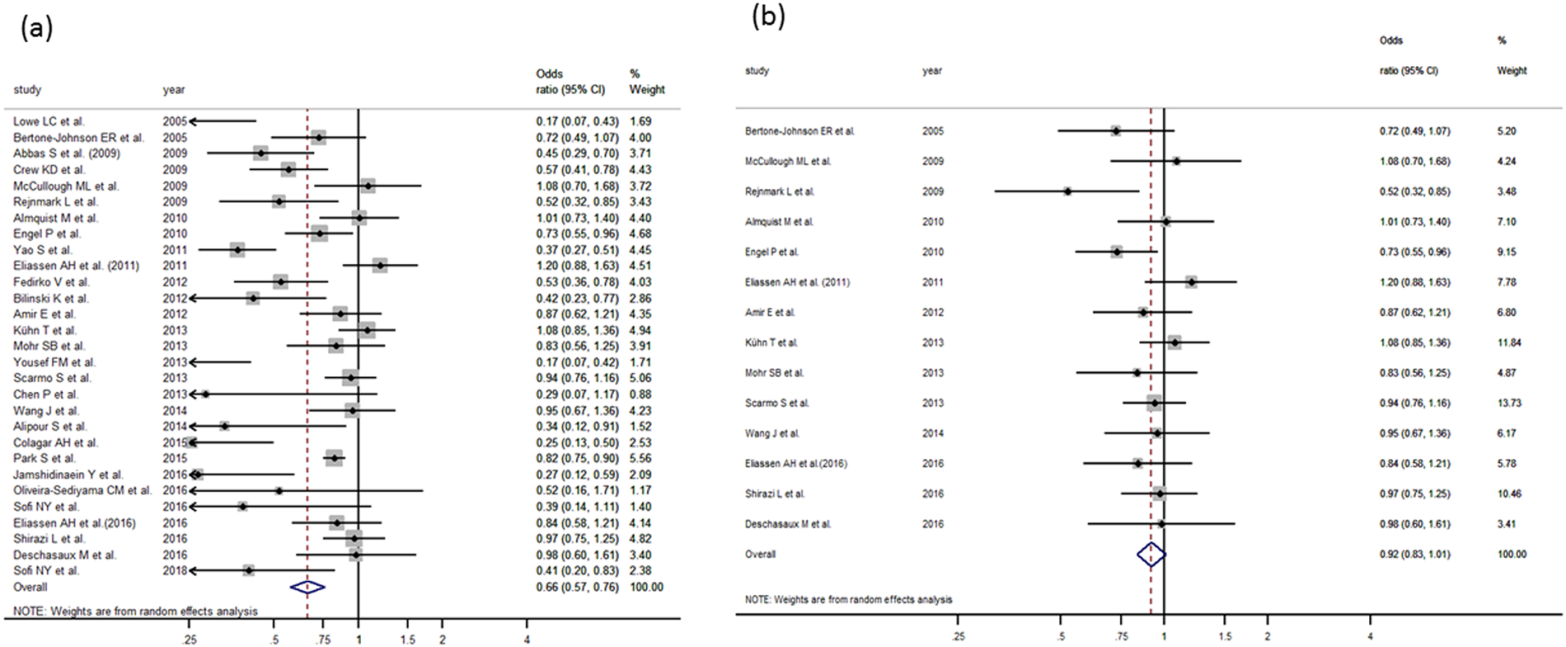
(**a**) Forest plot for the relationship between 25(OH)D and breast cancer in case control studies. (**b**) Forest plot for the relationship between 25(OH)D and breast cancer in nested case control studies.

Twenty-nine case control studies were analyzed to study the relationship between 25 (OH) D and breast cancer^10,19–22,25,27,29–35,38,42,44–46,48,49,51,55,56,58–63^ obtaining a pooled OR of 0.65 (95%CI: 0.56–0.76) (Fig. 2a, Table 4). This value was calculated using the random effects model because of the high heterogeneity (I^2^ = 77.76%) of the fixed-effect. Although Egger test cannot rule out a small-study effect (p = 0.001), no study shows a relevant influence. The funnel plot shows asymmetry (Supplementary Fig. 1a), indicating either publication bias or heterogeneity that cannot be explained by a random-effect meta-analysis. Rosenthal model shows that 1194 negative studies would be needed to lose statistical significance. In order to further clarify the heterogeneous result, we carried out a sensitivity analysis including only nested case-control studies^21,22,25,31–34,42,45,46,51,55,56,59^ reaching a pooled OR = 0.92 (95%CI: 0.83–1.01) (Fig. 2b) with I^2^ = 15.87%, Q-based p value = 0.22 and a very symmetrical-looking funnel plot (Supplementary Fig. 1b).

**Table 4:** Results from the meta-analysis.

Exposition	Group (Number of studies)	Type of study	OR/RR (95% CI)	I^2^
25(OH)D	All (n = 29)	Case-control	0.65 (0.56–0.76)	40.87%
	All (n = 4)	Cohort	0.85 (0.74–0.98)	3.56%
	ER+ (n = 5)	Case-control	0.98 (0.85–1.13)	0%
	ER– (n = 5)	Case-control	0.86 (0.64–1.15)	15.60%
	Postmenopausal (n = 19)	Case-control	0.74 (0.59–0.93)	13.16%
	Postmenopausal (n = 3)	Cohort	1.15 (0.59–2.23)	8%
	Premenopausal (n = 9)	Case-control	0.63 (0.49–0.80)	8.37%
Dietary vitamin D	All (n = 8)	Case-control	0.91 (0.72–1.17)	30.73%
	All (n = 5)	Cohort	1.00 (0.93–1.07)	0%
	Postmenopausal (n = 4)	Case-control	0.78 (0.68–0.90)	0%
	Postmenopausal (n = 5)	Cohort	0.95 (0.83–1.09)	19.13%
	Premenopausal (n = 5)	Case-Control	0.65 (0.52–0.82)	0%
	Premenopausal (n = 3)	Cohort	1.01 (0.86–1.18)	0%
Vitamin D supplements	All (n = 5)	Case-control	0.78 (0.63–0.98)	25.94%
	All (n = 2)	Cohort	1.06 (0.90–1.25)	0%
Total Vitamin D intake (dietary + supplements)	All (n = 4)	Case-control	0.84 (0.68–1.05)	18.65%
	All (n = 2)	Cohort	0.93 (0.86–1.02)	0%
	Postmenopausal (n = 5)	Cohort	0.94 (0.87–1.02)	17.64%
	Premenopausal (n = 3)	Cohort	0.90 (0.72–1.12)	10.83%

Four cohort studies^75,78–80^ provided results on 25(OH)D and breast cancer relationship, from which we obtained a pooled RR of 0.85 (95% CI:0.74–0.98).

**Figure 3:**
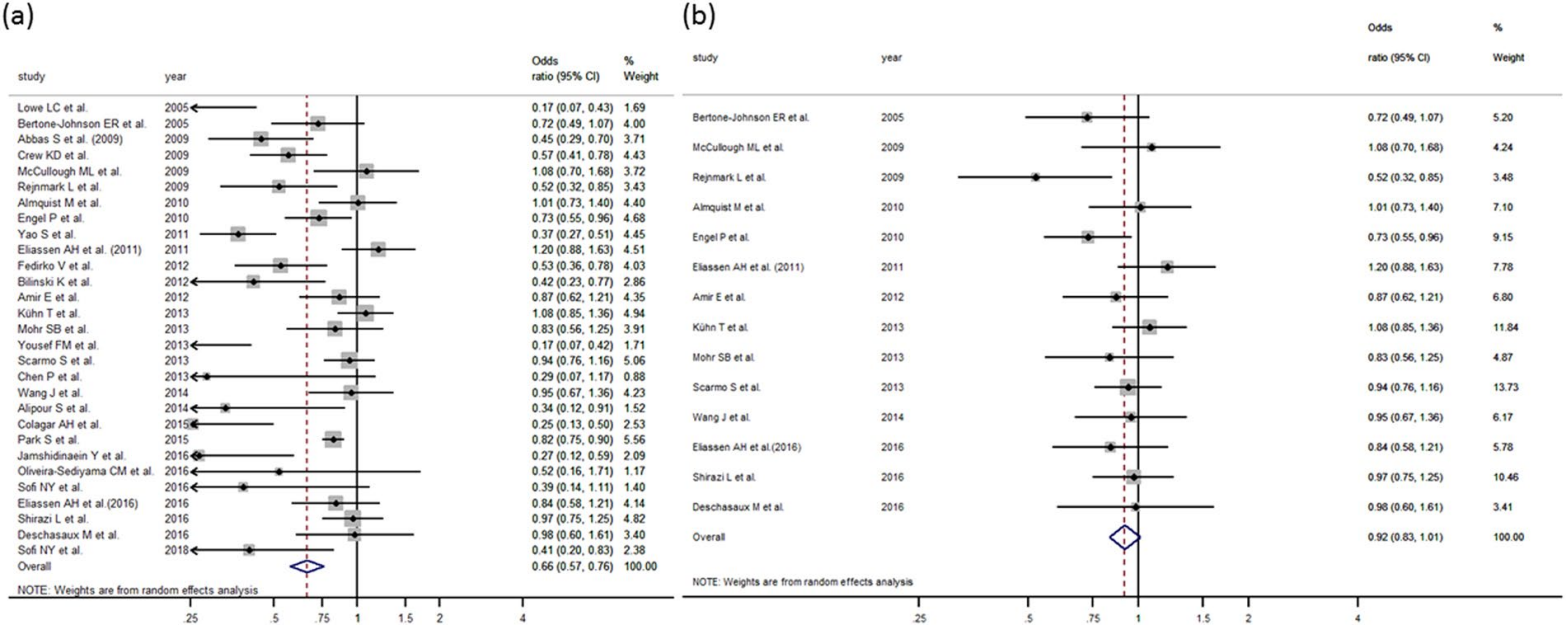
(**a**) Forest plot for the relationship between 25(OH)D and premenopausal breast cancer in case control studies. (**b**) Forest plot for the relationship between 25(OH)D and premenopausal breast cancer in nested case control studies.

We also analyzed the relationship between 25(OH) D and breast cancer, stratifying results by hormonal receptors (ER+/ER−) and menopausal status (postmenopausal or premenopausal). Regarding hormonal receptors (Table 4), we have found only one cohort study^80^ and five case-control studies^19,32,33,42,45^. In both cases (ER+ and ER− tumors) statistical significance was not reached. With respect to menopausal status (Table 4), we obtained a protective effect in both groups: nineteen case-control studies targeted postmenopausal women^18,21,28,30,34–36,38,41,47,49,51,55,60,81^ with a pooled OR of 0.74 (95%CI: 0.59–0.93), and nine focused on premenopausal^21,30,34,35,38,49,51,55,60^ obtaining a pooled OR of 0.63 (95%CI: 0.49–0.80) (Fig. 3a). When the sensitivity analysis was carried out including only nested case-control studies, the protective vitamin D – breast cancer association persisted only in the premenopausal group (Fig. 3b, Supplementary Table 1). On the other hand three cohorts studies analyzed separately postmenopausal women^79,81^ without reaching statistical significance (OR = 1.15 (0.59–2.23)).

### Relationship between 1,25(OH)2D and breast cancer

Three case-control studies^25,37,39^ examined the relationship between circulating 1,25(OH)2D and breast cancer; significant association was not found either in the whole analysis (pooled OR = 0.61 (0.33–1.16)) or in postmenopausal women (combined OR = 1.28 IC 95%: 0.98–1.67)^36,37^.

### Relationship between dietary vitamin D and breast cancer

We found eight case-control studies^24,38,40,50,52,53,57,64^ on the relationship between dietary vitamin D and breast cancer with a pooled OR of 0.91 (95%CI: 0.72–1.17) (Table 4, Supplementary Fig. 2a). In addition, by combining five cohort studies^66,68,70–72^ we obtained a RR of 1.00 (95% CI 0.93–1.07) (Table 4, Supplementary Fig. 2b).

When stratifying by menopausal status, four case-control^38,40,53,64^ and five cohort studies^66,73,74,76,77^ assessed the risk of breast cancer in postmenopausal women. The pooled OR for case-control studies was 0.78 (95%CI: 0.68–0.90) and the pooled RR for cohort studies was 0.95 (95%CI: 0.83–1.09) (Table 4). In both analyses, Egger test rejected the possibility of small study bias (p = 0.536 in case-control studies and p = 0.68 in cohort studies). On the other hand, five case-control studies^17,38,40,53,63^ and three cohort studies^66,73,77^ targeted premenopausal women; the pooled OR was 0.65 (95%CI: 0.52–0.82) for case-control studies and the RR for cohort studies was 1.01 (95% CI: 0.86–1.18) (Table 4).

### Relationship between supplements of vitamin D and breast cancer

We identified five case-control studies^23,24,43,52,65^ and two cohort studies^67,71^ that had evaluated the association between supplements of vitamin D and breast cancer risk. The pooled OR and RR were 0.78 (95% CI: 0.63–0.98) and 1.06(95% IC: 0.90–1.25) respectively (Table 4). Regarding menopausal status, Kim *et al*.^41^ published a study on five different populations of postmenopausal women; when combining all five results, we found no significant association (OR: 0.82 95%CI: 0.49–1.35).In addition, we found two case-control studies^26,54^ focused on premenopausal women obtaining a weak protection (pooled OR 0.89 95%CI (0.84–0.95)).

### Relationship between total vitamin D intake (dietary and supplements) and breast cancer

Finally, we found two cohort studies^69,71^ and four case control studies^23,24,38,64^ on vitamin D intake (dietary plus supplements) and breast cancer risk, providing no separate results on dietary/supplemented vitamin D origin. We obtained a combined RR = 0.93 (95% CI: 0.86–1.02) for cohort studies, and a combined OR = 0.84 (95% CI: 0.68–1.05) for case-control studies. Five cohort studies^69,73,74,76,77^ provided results on postmenopausal women (RR = 0.94 95% CI: 0.87–1.00) and three cohort studies^69,73,77^ on about premenopausal women (RR = 0.90 95% CI: 0.72–1.12) (Table 4). Only two case-control studies provided results according menopausal status^38,64^ without being significant in both groups.

## Discussion

According to our results, 25(OH)D levels were associated with smaller risk of breast cancer in both case-control and cohort studies; these results were consistent on premenopausal women for case-control studies but could not be analyzed for cohort studies. Results for the relationships between breast cancer and dietary vitamin D or between breast cancer and vitamin D supplements, however, showed a protective association only in case-control studies.

In relation to the influence of vitamin D on breast cancer development prospective (cohort and nested case-control) and case control studies tend to show discrepant results: case-control studies usually show a protective effect while prospective studies rarely find it^87^. This discrepancy might be the result of several factors: Firstly, it is well known that prospective studies are less prone to be affected by both information and reverse-causation bias. Secondly, several authors highlight the season when the vitamin D measurement was made as a potential limitation of case-control studies. Eliassen *et al*.^33^ in a nested case-control study found an inverse association between serum 25(OH) D levels and breast cancer limited only to summer measures. It can be assumed that people with low vitamin D levels in summer would also have low levels year-round; therefore, vitamin D levels in summer would be more adequate for analyzing vitamin D – breast cancer relationship than vitamin D levels in any other moment of the year.

When stratifying by menopausal status, our meta-analysis shows a consistent protective effect of 25(OH) D in both case-control and nested case-control studies, but only in premenopausal women. There are different explanations for the influence of menopausal status in the relationship between vitamin D and breast cancer. One of them may be related to the joint relationship between vitamin D and insulin-like growth factors (IGFs). IGF-I is a mitogenic and antiapoptotic peptide that can stimulate the proliferation of breast epithelial cells, increasing the risk of neoplastic transformation^88,89^. The active vitamin D metabolite is able to block the mitogenic effects of IGF-I, leading to a decrease in proliferation and an increase in apoptosis^90^. As there is a physiological decline of the IGF with aging^91^, the interaction between IGF pathways and vitamin D is likely to be stronger for premenopausal than for postmenopausal women, leading to greater risk reduction in premenopausal breast cancer^73,92^. Finally, high levels of vitamin D may reduce progesterone and estradiol, providing a potential mechanism for reducing breast cancer risk in young women^93^.

Previous meta-analyses of prospective studies showed contradictory results. Kim *et al*.^13^ (who included 24 studies, 14 of those having measured serum 25(OH)D) found a slightly stronger inverse association among premenopausal than among postmenopausal women but without significant differences, whereas in the meta-analysis of Bauer *et al*.^8^ (nine studies included) the inverse association was only observed in postmenopausal women. In our meta-analysis, new prospective studies^31,33,41,56,58,59,67,78–81,94^ not included in previous reviews, were added and this fact may explain the differences in the results.

Concerning hormonal receptors (ER+/ER−), the relationship with breast cancer remains controversial. On the one hand, a decreased risk in ER+ would be expected, since it seems that sensitivity to 1,25(OH)2D is generally reported as being higher in breast cancer cells that express the estrogen receptor than in those that do not^93,95^. It has been demonstrated that treating breast cancer cells ER+ with 1,25(OH)D3 induces a cell cycle shutdown in GO/G1^3,96^. On the other hand, two-thirds of triple negative tumors express VDR^97^ and it has been demonstrated that VDR expression is inversely associated with more aggressive breast cancer^98^. In consonance with previous epidemiological studies^32,33,42,45^, our study does not reach significant differences when the analysis was performed separately in ER+ or ER− subgroups. However, other studies found a decreased risk of ER− breast cancer regarding the serum levels of 25 (OH) D^18,60^.

No relationship is found between the level of circulating 1,25(OH)2D and breast cancer. This result is consistent with previous studies^9^, while Janowsky *et al*.^39^ found an inverse association. Several authors consider that 1,25(OH)2D is not a good indicator of vitamin D status: First, 1,25(OH)2D’s half-life is only 4–6 h, whereas 25(OH)D’s half-life is 3 weeks; second, 1,25(OH)2D is influenced by many factors^10^, for instance, it can be elevated in patients with vitamin D deficiency as a result of hyperparathyroidism^12,99^; finally, as 1,25(OH)2D is metabolized by 1-α -hydroxylase in breast tissue, plasma levels may not adequately represent breast tissue levels^12,100^.

We do not find a relationship between vitamin D intake and breast cancer in the overall analysis. In contrast, when stratifying by menopausal status, a protective effect is observed in case-control studies in both premenopausal and postmenopausal women, whereas this association is not present in cohort studies. On the other hand, when analyzing the influence of vitamin D supplements on breast cancer risk, we find a borderline protective effect.

In the relationship between vitamin D intake (dietary and/or supplements) and breast cancer, most observational studies showed non-significant differences; only two articles^17,53^ found a protective association. In a previous meta-analysis^13^, this association was not significant for either vitamin D intake or supplements.

A probable explanation for the lack of association observed in the analysis of dietary intake or supplements compared to the 25(OH)D levels may be that the main source of vitamin D is sunlight rather than food or supplements.

In addition, the French E3N Cohort Study^12^ reported that high vitamin D intake is associated with lower breast cancer risk in regions with high ultraviolet solar radiance. These results suggested that the total amount of vitamin D needed to reach a protective effect on breast cancer is too high to be achieved in regions with low ultraviolet radiance. Under these circumstances, as the vitamin D intake has to be higher than the usually recommended, it could eventually lead to side effects such as hypercalcemia, constipation or muscle weakness.

Our study has some limitations; firstly each article uses different cutoff points according to serum levels of vitamin D. To analyze it we restricted our analysis to the comparison between the highest vs. lowest category of exposure. This analysis strategy does not allow for a dose-response analysis. Moreover, we carried out a sensitivity analysis excluding one study at a time, showing that no single study substantially affected the pooled RR/OR. Secondly, there is huge variability in the literature on the type of vitamin D studied, which makes it difficult to perform the analysis. In addition, levels of vitamin D depend on the season, so it would be advisable to take all samples at the same time, or at least refer to when they were collected^75^. Thirdly, case-control studies are more prone to methodological issues, such as recall and selection biases, which limits the strength and quality of evidence. However, about half of the case-control studies included in our meta-analysis are nested in cohort studies, which minimizes the possibility of introducing biases. Finally, breast cancer is a heterogeneous disease and it is possible that vitamin D only affects certain breast cancer subtypes. However, this aspect has been scarcely studied in primary articles, so we have not been able to analyze it in the present meta-analysis.

Despite these limitations, our study also has several strengths; first, we have gathered all the observational studies published in the last twenty years. In addition, we have focused the analysis on different types of vitamin D exposure (diet, supplements and blood-levels of 25(OH) D and 1,25(OH)2D) whereas other meta-analyses are only focused on 25(OH)D levels^9,10,16,99^ or vitamin D intake^12^. This strategy allows us to obtain a more detailed analysis of the relationship between vitamin D and breast cancer.

In conclusion, our meta-analysis supports the hypothesis that high serum levels of 25(OH) vitamin D has a protective effect on breast cancer risk in premenopausal women; we cannot draw the same conclusion regarding vitamin D intake or supplements of vitamin D since the number of studies are still limited and publication biases cannot be excluded.

## Electronic supplementary material

Supplementary material
