# Supplementary material for: Vitamin D exposure and Risk of Breast Cancer: a meta-analysis
Source: Sci Rep. 2018 Jun 13;8:9039. doi: 10.1038/s41598-018-27297-1 (PMC5997996; doi:10.1038/s41598-018-27297-1)

Full title: Vitamin D exposure and Risk of Breast Cancer: a meta-analysis

Short title: Vitamin D exposure and breast cancer

Authors:

Nuria Estébanez [1], Inés Gómez-Acebo[1,2], Camilo Palazuelos[1,2], Javier Llorca[1,2], Trinidad Dierssen-Sotos[1,2].

Affiliations:

1: University of Cantabria – IDIVAL, Santander, Spain.

2: CIBER Epidemiología y Salud Pública (CIBERESP), Madrid, Spain

Corresponding author:

Trinidad Dierssen-Sotos

Medicina Preventiva y Salud Pública

Facultad de Medicina

Avda. Herrera Oria s/n

39011 Santander

Cantabria

Phone #34-942201993/ Fax #34-942201903

E-mail: dierssent@unican.es

Supplementary table 1. - Results of global case-control studies versus nested case-control studies on the relationship between vitamin D and breast cancer

Exposition	Group	OR case-control and nested case control studies. Number of studies included	OR nested case-control studies. Number of studies included
25(OH)D	All	0.65 (0.56-0.76) N=*29*	0.92 (0.84-1.01) *N=14*
	ER+	0.98 (0.85-1.13) *N=5*	0.98 (0.85-1.13) *N=4*
	ER -	0.86(0.64-1.15) *N=5*	0.94 (0.76-1.18) *N=4*
	Postmenopausal	0.74 (0.59-0.93) *N=19*	0.97 (0.82-1.14) *N=12*
	Premenopausal	0.63(0.49-0.80) *N=9*	0.67 (0.49-0.92) *N=4*
Dietary vitamin D	All	0.91 (0.72-1.17) *N=8*	2.79(0.85-9.15) *N=1*
	Postmenopausal	0.78(0.68-0.90) *N=4*	NO STUDIES
	Premenopausal	0.65(0.52-0.82) *N=5*	NO STUDIES
Vitamin D supplement	All	0.78(0.63-0.98) *N=5*	NO STUDIES
	Postmenopausal	0.69 (0.34-1.41) *N=2*	0.82 (0.49-1.35) *N=5 (five populations in 1 article)*
Total Vitamin D intake (dietary + supplements)	All	0.84 (0.68-1.05) *N=4*	NO STUDIES

Supplementary fig. 1a. Funnel plot for cases control studies 25(OH)D and breast cancer

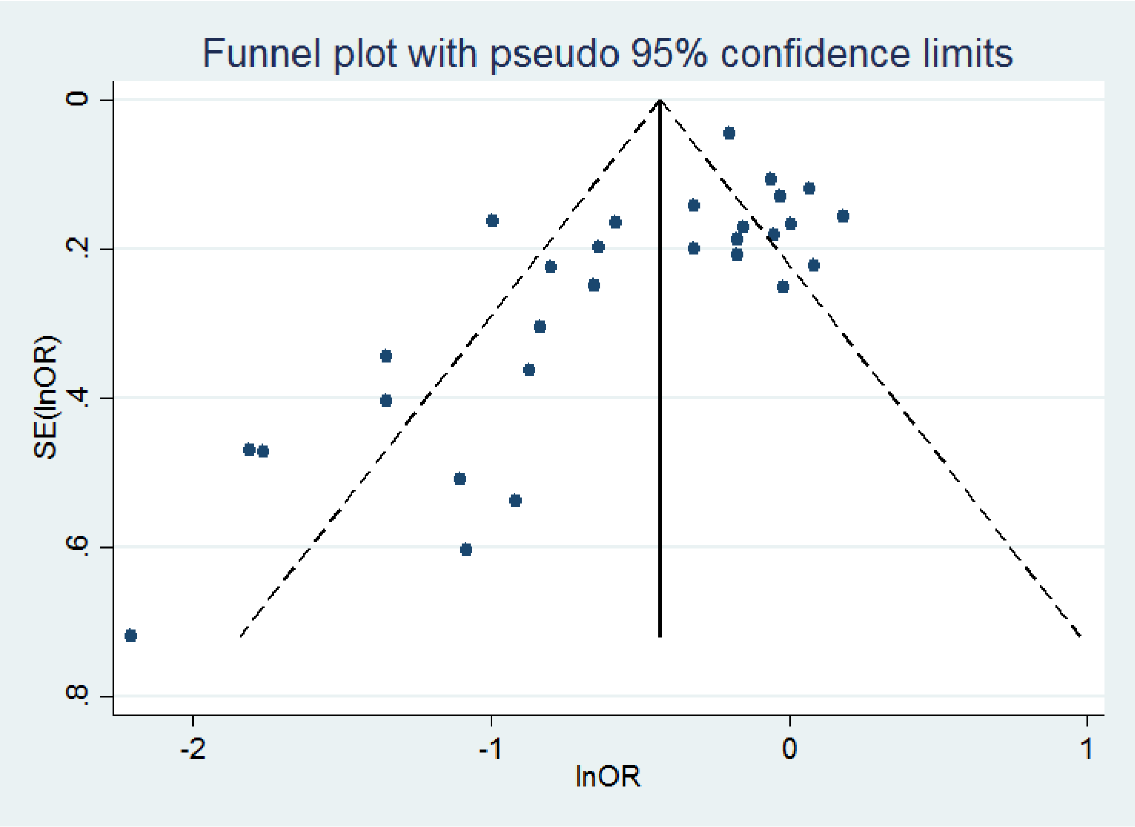

Supplementary fig. 1b. Funnel plot for nested cases control studies 25(OH)D and breast cancer

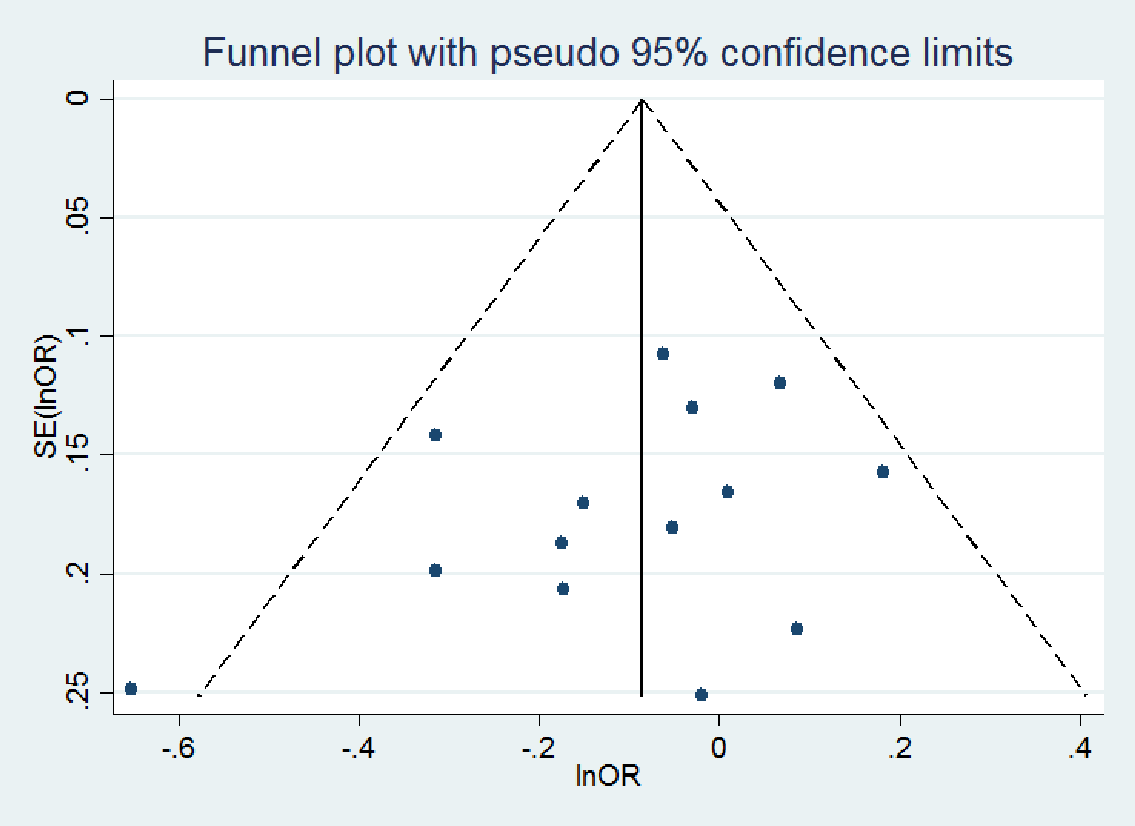

Supplementary fig. 2a. Forest plot for the relationship between dietary vitamin D and breast cancer in case control studies

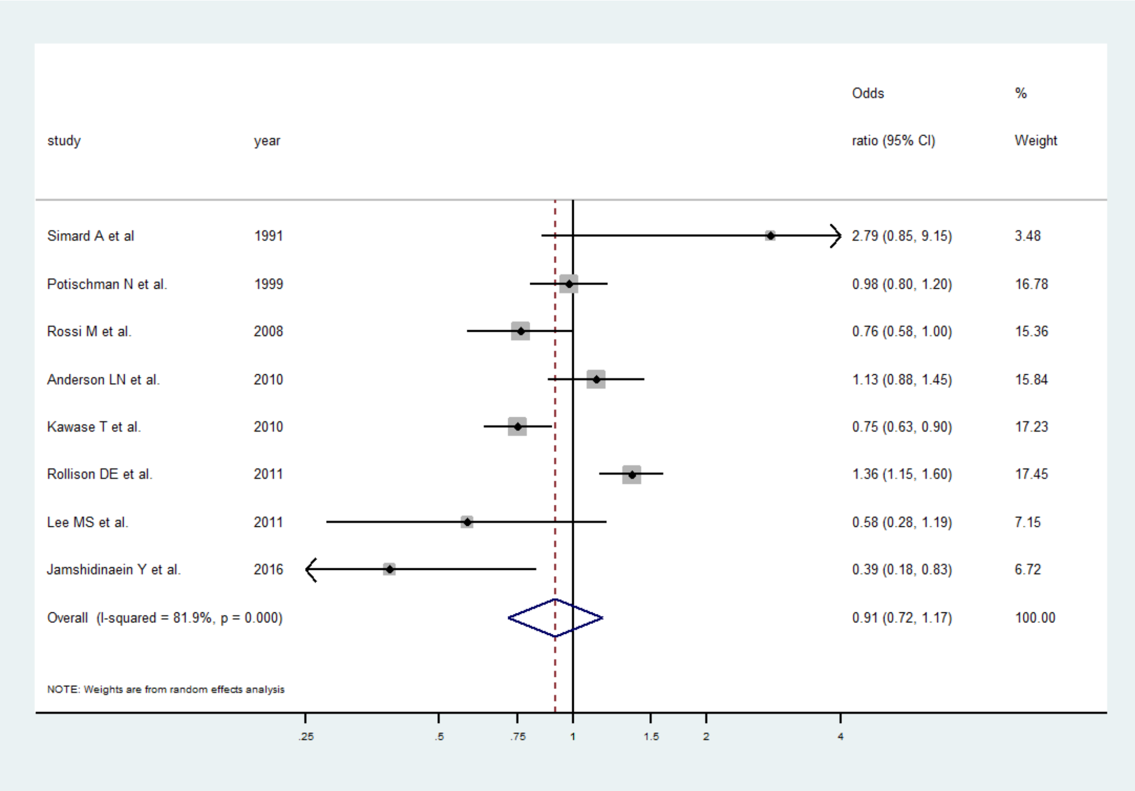

Supplementary fig. 2b. Forest plot for the relationship between dietary vitamin D and breast cancer in cohort studies

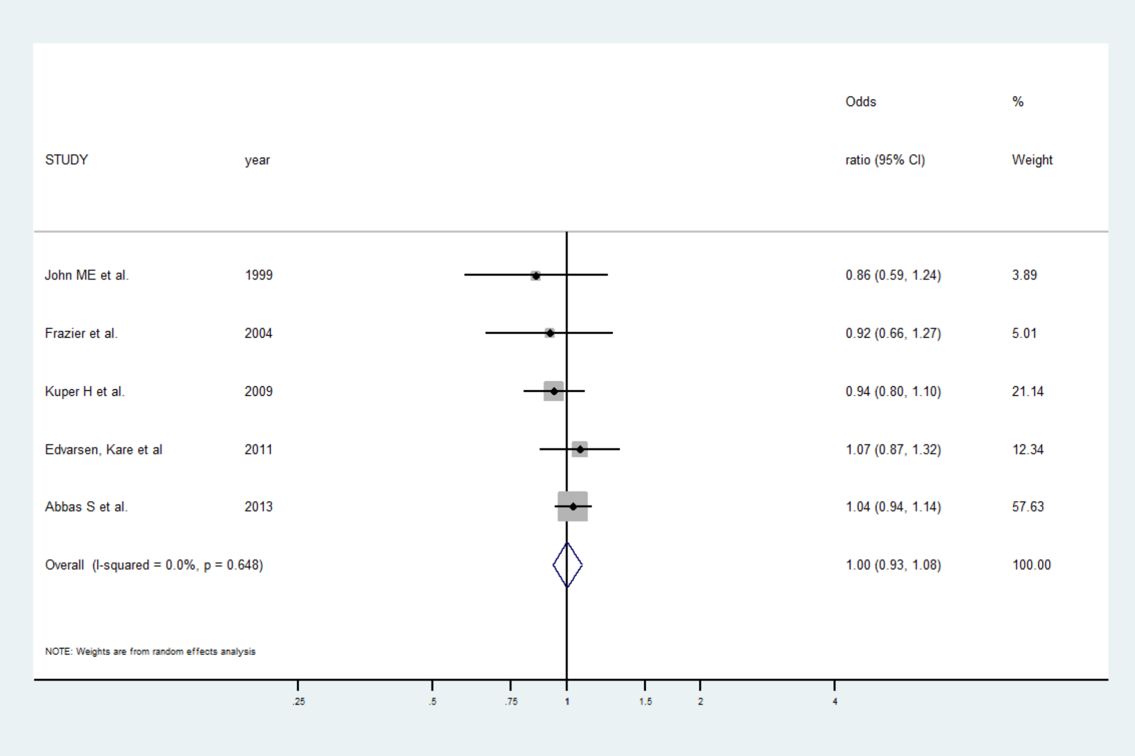

Supplement: Supplementary file 1 — Supplementary material [file 41598_2018_27297_MOESM1_ESM.pdf]
